# The Drosophila Fry protein interacts with Trc and is highly mobile *in vivo*

**DOI:** 10.1186/1471-213X-10-40

**Published:** 2010-04-20

**Authors:** Xiaolan Fang, Qiuheng Lu, Kazou Emoto, Paul N Adler

**Affiliations:** 1 Biology Department, Cell Biology Department, Cancer Center, Morphogenesis and Regenerative Medicine Institute, University of Virginia, Charlottesville, VA 22903, USA; 2 Neural Morphogenesis Laboratory, National Institute of Genetics, Yata 1111, Mishima, Shizuoka 411-8540, Japan; 3 Howard Hughes Medical Institute, Departments of Physiology, Biochemistry, and Biophysics, University of California San Francisco, San Francisco, CA 94143, USA

## Abstract

**Background:**

Cell polarity is a common feature of eukaryotic cells. The NDR kinases have been found to regulate polarized growth in both animal cells and fungi. Drosophila Tricornered is an NDR kinase that is essential for the normal polarized growth of extensions of epidermal cells and for the tiling and branching of dendrites of da sensory neurons. Tricornered function requires interacting with the large Furry protein (3479 amino acid).

**Results:**

We constructed a *furry *(*fry*) transgene and established that it rescued the lethality of *fry *null mutations. The encoded protein was tagged at both its amino and carboxy termini and this allowed us to demonstrate that the protein existed as an uncleaved protein in vivo. We used the C terminal GFP tag to follow the protein in vivo and found it to be highly mobile. Interestingly Fry accumulated at the distal tip of growing bristles. We established that Fry and Trc could be co-immunoprecipitated from wing discs.

**Conclusions:**

The mobility of Fry in both bristles and dendrites suggests that it could function in directing/mediating the intracellular transport needed for polarized growth. Our observations that full length Fry and Trc show only partial co-localization in growing bristles while an amino terminal fragment of Fry shows close to complete co-localization with Trc suggests that the interaction between these proteins is transient and regulated.

## Background

### NDR kinase module

NDR (Nuclear Dbf2 related) kinases are members of a conserved subfamily of serine/threonine kinases, which regulate polarized growth, cell division, cell morphology, centrosome duplication, neural outgrowth and dendritic tiling and branching [1-11]. These kinases function in association with conserved protein partners and together these protein complexes represent functional modules.

*tricornered *(*trc*) encodes the *Drosophila *Ndr kinase. Mutations in this gene are recessive lethal and have phenotypes in both the epidermis (typically studied in genetic mosaics) and in sensory neurons [4,12]. In the epidermis *trc *mutations result in dramatic split and clustered hair and split bristle and arista lateral phenotypes [12]. In dendritic arborization (da) sensory neurons *trc *mutations result in increased dendrite branching and a failure in dendrite tiling [4]. Related phenotypes are seen in other species with mutations in NDR kinases. A similar dendrite phenotype is associated with mutations in the *C. elegans trc *homolog *sax-1 *[8]. Polarized growth defects are seen in a variety of fungi with NDR mutations. In *S. cerevisiae CBK1 *(the *trc *homolog) mutations result in rounder than normal cells due to extended isotropic growth and a failure in cell separation due to a failure of the bud initiating the daughter cell gene expression program [1,2,9]. In *S. pombe*, *orb6 *(the *trc *homolog) mutations result in round instead of rod shaped cells [6] and in Neurospora mutations in *cot-1 *lead to increased hyphal branching [13,14]. In mammalian cells NDR kinases have been shown to regulate centriole duplication and the alignment of chromosomes on the mitotic spindle [15,16].

NDR kinases such as Trc, SAX-1, Dbf2p, Cbk1p and Orb6p function in complexes with both Mob and Fry family proteins [2-4,7,8,10,11,17-22]. These interactions have been found to be essential for kinase function *in vivo *and for kinase activity *in vitro*. The *fry *gene of Drosophila was the founding member of this family and it encodes a protein of 3479 amino acids [23]. Mutations in *fry *lead to similar phenotypes to *trc *in both the epidermis and in da neurons [4,12]. Similarity to the mammalian homologs extends over more than 3000 amino acids with the most highly conserved region located in the amino terminal 1/3 of the protein. This region is more than 600 amino acids long (60% identity from human to fly) and is found in all Fry family proteins [23]. The very large size of these proteins makes them difficult to study by biochemical approaches and also suggests that they might function as a scaffold to bring together other proteins. A recent exciting result is that the mammalian Fry was found to be a microtubule binding protein [24].

### Subcellular localization and movement of Fry

We previously observed that Fry had a punctate distribution in growing wing hairs [10]. We extended those experiments and found that Fry was also punctate in developing bristles. Interestingly, we observed the preferential accumulation of Fry at the distal tip of growing bristles. To facilitate further studies on Fry we constructed a transposon where a *fry *minigene was under UAS control. When the expression of this gene was driven by *actin-Gal4 *we obtained complete rescue of the recessive lethality of *fry *and close to complete rescue of the multiple hair cell phenotype on the wing. The Fry protein encoded by this transgene was tagged on its amino terminus by Myc_6 _and on its carboxyl terminus by GFP. The double tag enabled us to determine that the protein existed as a complete uncleaved protein in vivo. The GFP tag allowed us to visualize Fry in living cells. FRAP (fluorescence recovery after photobleaching) experiments demonstrated that Fry was highly mobile in both developing sensory bristle shafts and in dendrites of da neurons.

### Regulation of Trc-Fry interaction

Previously we found that endogenous Fry and Trc did not extensively co-localize in pupal wing cells or bristles [10]. This was also the case for transgene encoded Fry and Trc. Interestingly we found extensive co-localization between Trc and an amino terminal fragment of Fry that mediated the co-immunoprecipitation of the two proteins [10]. Finally, we used the two-hybrid system to identify a small region of the Trc protein that is essential for interacting with Fry.

## Results

### Construction and characterization of a *fry *rescue transposon

The *fry *mRNA is predicted to be 10873 bp and much of it was not recovered in the Drosophila genome project cDNA collection. We assembled a *fry *minigene using a 5' fragment synthesized *de novo*, two middle fragments of cDNA derived from mRNA by RT-PCR and a 3' fragment of genomic DNA generated by PCR that contained 5 exons and 4 introns (see Methods). This minigene was subcloned into the pTWG vector and used to generate transgenic flies. The final transgene was tagged at its 5' end by six copies of the Myc epitope tag and at its 3' end by GFP.

**Figure 1:**
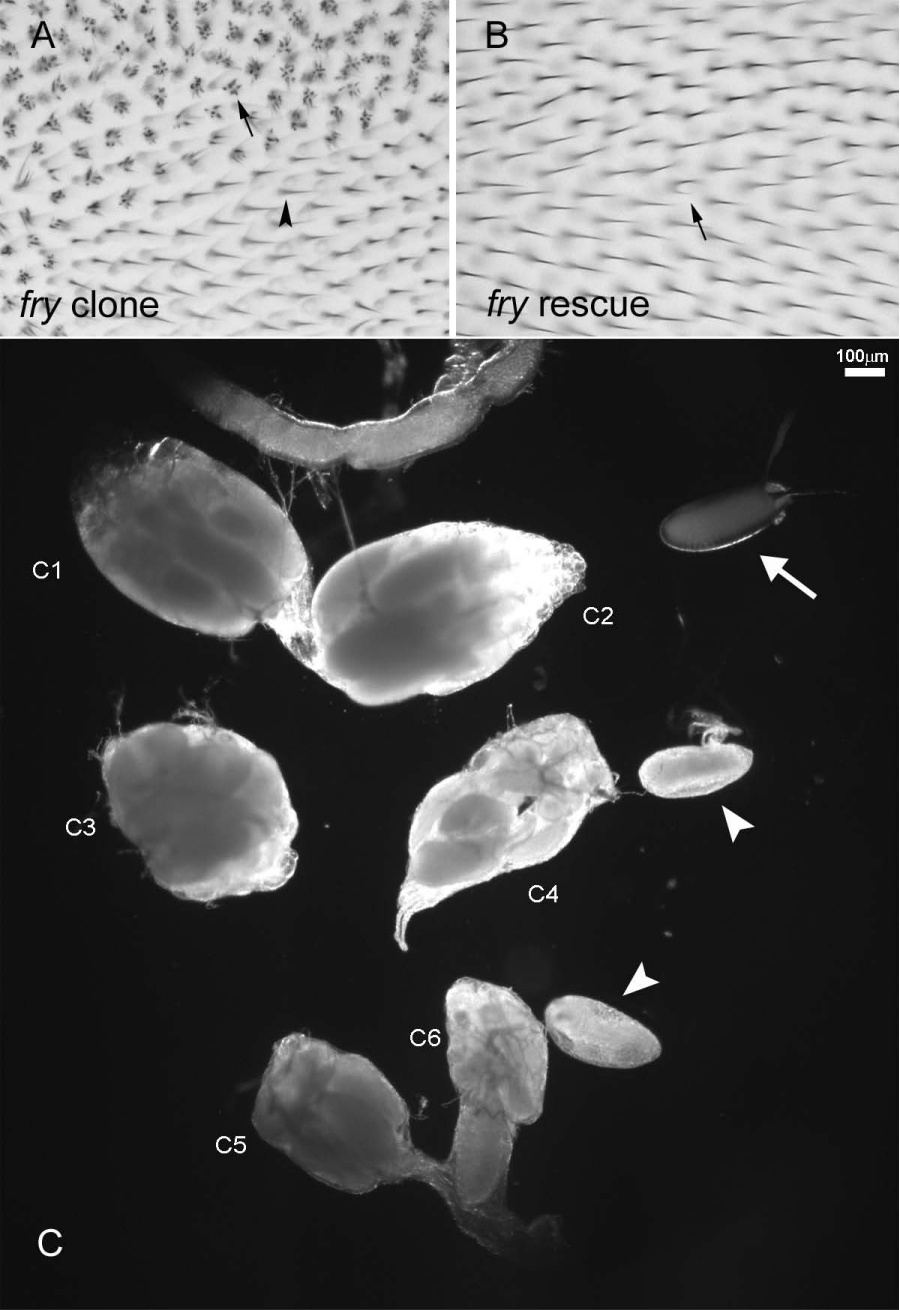
**Myc-Fry-GFP expression can rescue the mutant phenotype caused by a loss of function *fry *mutation**. (A) An adult wing containing a *fry *loss of function mutation clone. Arrow points to a *fry *mutant cell. Arrowhead points to a wild type cell. (B) Rescue of the *fry *wing hair phenotype. The genotype of the rescued fly is *UAS-Myc-fry-GFP/actin-GAL4; fry*^1^/*fry*^2^. Arrow points to the single cell in the field that shows a weak multiple wing hair phenotype. All other cells show complete rescue. (C) Ovaries from wild type and rescued adult flies. C1 and C2 were a pair of ovaries from a wild type female (OreR). C3-C6 were ovaries from rescued female flies (*actinGAL4/UAS-Myc-fry-GFP; fry*^1^/*fry*^2^). C3 and C4 were a pair, and C5 and C6 were a second pair. Note that the ovaries from the rescued flies are smaller than normal. The arrow points to a wild type egg and arrowheads point to mutant eggs that are rounder than normal.

To determine if the transgene encoded a functional protein we crossed *UAS-myc-fry-GFP/CyO; fry*^1^/*TM6 *and *actin-gal4; fry*^2^/*TM6 *flies. The resulting *UAS-myc-fry-GFP/actinGAL4; fry*^1^/*fry*^2^flies eclosed at the expected frequency (98.4%, 62/63 observed/expected). Thus, the transgene provided complete rescue of the lethality seen in *fry*^1^/*fry*^2 ^flies (both of these alleles are likely null alleles). We examined adult wings from these flies and found that more than 99% of wing cells produced a single hair compared to the more than 4 hairs per cell seen with *fry *mutants (Figure 1A, B). The bristle phenotype of *fry *was completely rescued. Thus, the Myc and GFP tags did not interfere with the protein's function. In contrast, the *UAS-myc-fry-GFP/actinGAL4; fry*^1^/*fry*^2 ^flies were both male and female sterile. This phenotype had 100 percent expressivity and penetrance. The paired ovaries of rescued females were often of different sizes, and the eggs produced were rounder than normal and were rarely laid (Fig. 1C). We suggest that the transgene was not expressed well in the ovary and that *fry *has an important function in egg morphogenesis.

The *act-Gal4 *driver used in the rescue experiments does not drive a high level of expression in most cell types. When we drove expression with stronger drivers such as *ptc-Gal4 *or *ap-Gal4 *the resulting animals appeared relatively normal, with the exception that driving expression using *ap-Gal4 *led to an occasional loss of thoracic macrochaetae. This is also seen in flies that carry *ap-Gal4 *without *UAS-myc-fry-GFP *although at a lower frequency. We observed an average of 4 dorsocentral bristles in wild type flies (n = 26) and *UAS-myc-fry-GFP *flies (n = 24), 3.8 (SEM = 0.038) in *ap-Gal4 *flies (n = 122) and 3.01 (SEM = 0.1) in flies that contained both *ap-Gal4 *and *UAS-myc-fry-GFP *(n = 86). The difference between the *ap-Gal4 *and *ap-Gal4/+; UAS-myc-fry-GFP *flies was highly significant (p < 0.001, Mann-Whitney Rank Sum test). On the whole however, our observations argue that increased Fry levels did not seriously affect most fly cells.

### The Fry protein is intact in vivo

**Figure 2:**
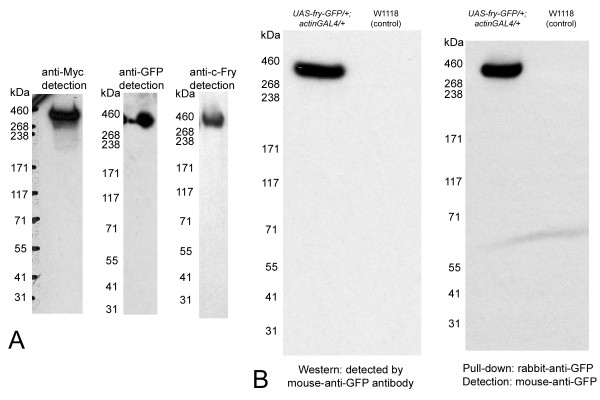
**Detection of intact Fry**. (A) A single and very large protein is detected in Western blots of *UAS-Myc-fry-GFP/+; actinGAL4/+ *tissues probed with anti-Myc, anti-GFP or anti-c-fry antibody. The protein band migrates between marker proteins that are 268 kDa and 460 kDa. Consistent with its predicted molecular weight (418 kDa) the tagged Fry protein migrates to closer to the 460 kDa marker. (B) Fry can be detected after immunoprecipitation. The left panel shows a Western blot of extracts of 30 wing discs from *UAS-Myc-fry-GFP/+; actin-GAL4/+ *larvae or *w*^1118 ^larvae (control lane) detected by anti-GFP antibody. A strong Fry signal is seen in the experimental lane but not in the control lane. The right panel shows similar extracts immunoprecipitated with rabbit anti-GFP antibody followed by detection using mouse anti-GFP antibody. The light line at the lower part of the film is caused by bending of the film and is not a western signal. Note the strong positive signal in the *UAS-Myc-fry-GFP/+; actin-GAL4/+ *lane showing that we were able to immunoprecipitate the large Fry protein.

The Myc-Fry-GFP protein encoded by the transgene was characterized by Western blot analysis. When detected using anti-GFP antibody (Figure 2A, B), the protein ran close to the predicted size although the very large size of the protein (predicted to be 418 kDa) makes precise molecular weight estimation difficult. We confirmed that this protein contains the C-terminal Fry sequence by probing a separate Western blot using an antibody directed against the C terminal end of the endogenous Fry protein (Figure 2A) [10]. To confirm that the Myc-Fry-GFP protein is present as a single protein *in vivo*, we also probed paired Western blots with anti-Myc or anti-GFP antibodies. Both antibodies recognized a common band of approximately 400 kDa (Figure 2A) indicating that the protein was intact in vivo. We also found that we could pull down the full length Myc-Fry-GFP protein using a polyclonal rabbit-anti-GFP antibody and detect it using mouse-anti-GFP antibody (Figure 2B). This result opens up the possibility for additional experiments to study the biochemistry of Fry.

### Fry Protein localization

**Figure 3:**
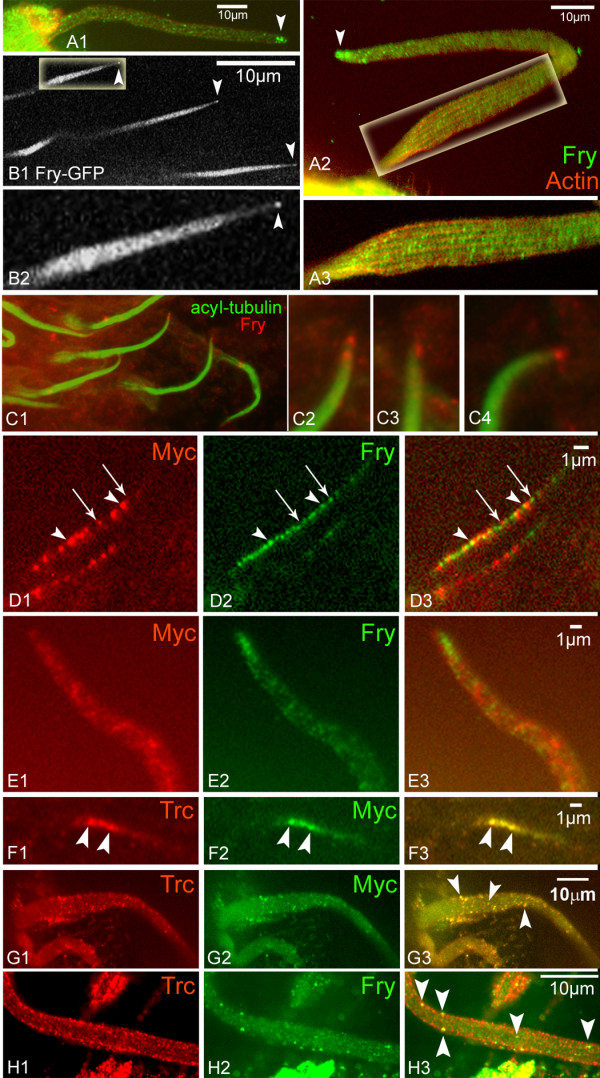
**Intracellular localization of Fry in bristles and hairs**. (A1) A wt bristle stained with anti-Fry antibody shows punctate localization and accumulation at the tip (arrowhead). (A2) A second bristle and a blow up (A3). (B1) Thoracic microchaetae from a living *UAS-Myc-fry-GFP/+*; *neur-gal4/+ *pupae. Note the tip accumulation of Myc-Fry-GFP (arrowheads). B2 higher magnification of B1 (C) C1 is from a pupal head stained with anti-acyl-tubulin and anti-Fry. Endogenous Fry accumulates at the tip. Blow ups of bristle tips are shown in C2-5. Similar results were obtained using anti-tubulin antibodies. (D) Growing wing hairs from a *UAS-Myc-N-fry/+; actinGAL4/+ *pupal wing immunostained with both anti-Myc and anti-C terminal Fry antibodies. Both types of Fry protein show a punctate distribution but the two only partly co-localize (arrowheads). Most puncta contain only one protein (arrows). (E) The distal half of a bristle from a *UAS-Myc-N-fry/+; actinGAL4/+ *pupa immunostained with both anti-Myc and anti-C terminal Fry antibodies. Note the distal accumulation of endogenous Fry but not Myc-N-Fry. (F) A growing wing hair from a *UAS-Myc-N-fry/+; actinGAL4/+ *pupal wing immunostained with both anti-Trc and anti-Myc. Note the extensive co-localization between Trc and Myc-N-Fry. Arrowheads point to two puncta that stain for both. (G) A growing bristle from a *UAS-Myc-N-fry/+; actinGAL4/+ *pupa immunostained with both anti-Trc and anti-Myc. Note the extensive co-localization. Arrowheads point to three puncta that stain for both. (H) A bristle from a wt pupa stained with both anti-Trc and anti-Fry antibodies. Note that only a few puncta stain with both antibodies (arrowheads).

We previously found the endogenous Fry protein had a punctate distribution in developing wing hairs [10]. When we examined Fry in growing bristles by immunostaining, we also found it to be punctate (Figure 3A3). In addition, we found Fry enriched at the distal tips of growing bristles (Figure 3A1, A2). We observed this for bristles on the head, thorax and wing and in both macrochaetae and microchaetae. We had previously noted that there was only modest co-localization of Trc and Fry in hairs [10] and we found this to also be the case in bristles (Figure 3H). Only a minority of Trc staining puncta also stained positively for Fry and similarly only a minority of Fry staining puncta also stained positively for Trc. There was no evidence of co-localization of Fry with either the actin or microtubule cytoskeletons (Figure 3A3, C1-4). Growing bristles stained intensely with an anti-acylated tubulin antibody suggesting that many bristle microtubules are stable.

We expressed the *UAS-myc-fry-GFP *transgene in bristle cells using the *neur-GAL4 *driver and found that we could also observe the distal tip accumulation of Myc-Fry-GFP in thoracic microchaetae in vivo (Figure 3B1, B2). Surprisingly the distal tip enrichment of transgene-encoded protein was not obvious in macrochaetae in either in vivo imaging or immunostaining experiments. In previous experiments we had observed that the *neur-Gal4 *drives a higher level of target gene expression in macrochaetae than microchaetae, thus we suspected that the failure to observe distal enrichment could be due to high level of expression saturating tip binding sites. To test this hypothesis we generated *UAS-myc-fry-GFP/Tub-Gal80*^*ts*^*; neur-Gal4/+ *animals and grew these at 21°C. At this temperature the Gal80^ts ^protein is active and blocks the ability of Gal4 to drive expression from the transgene. We collected white prepupae (WPP), aged these at 21°C for 48 hrs at 25°C and then moved these to 29°C for 1.5-2 hrs to briefly induce the expression of the transgene. This resulted in a much lower level of Myc-Fry-GFP accumulation (as judged by immunostaining) than in previous transgene experiments and in these preparations we could detect enrichment of Myc-Fry-GFP at the distal tips of some bristles (data not shown). We concluded that the tags did not alter the subcellular distribution of the Fry protein.

**Figure 4:**
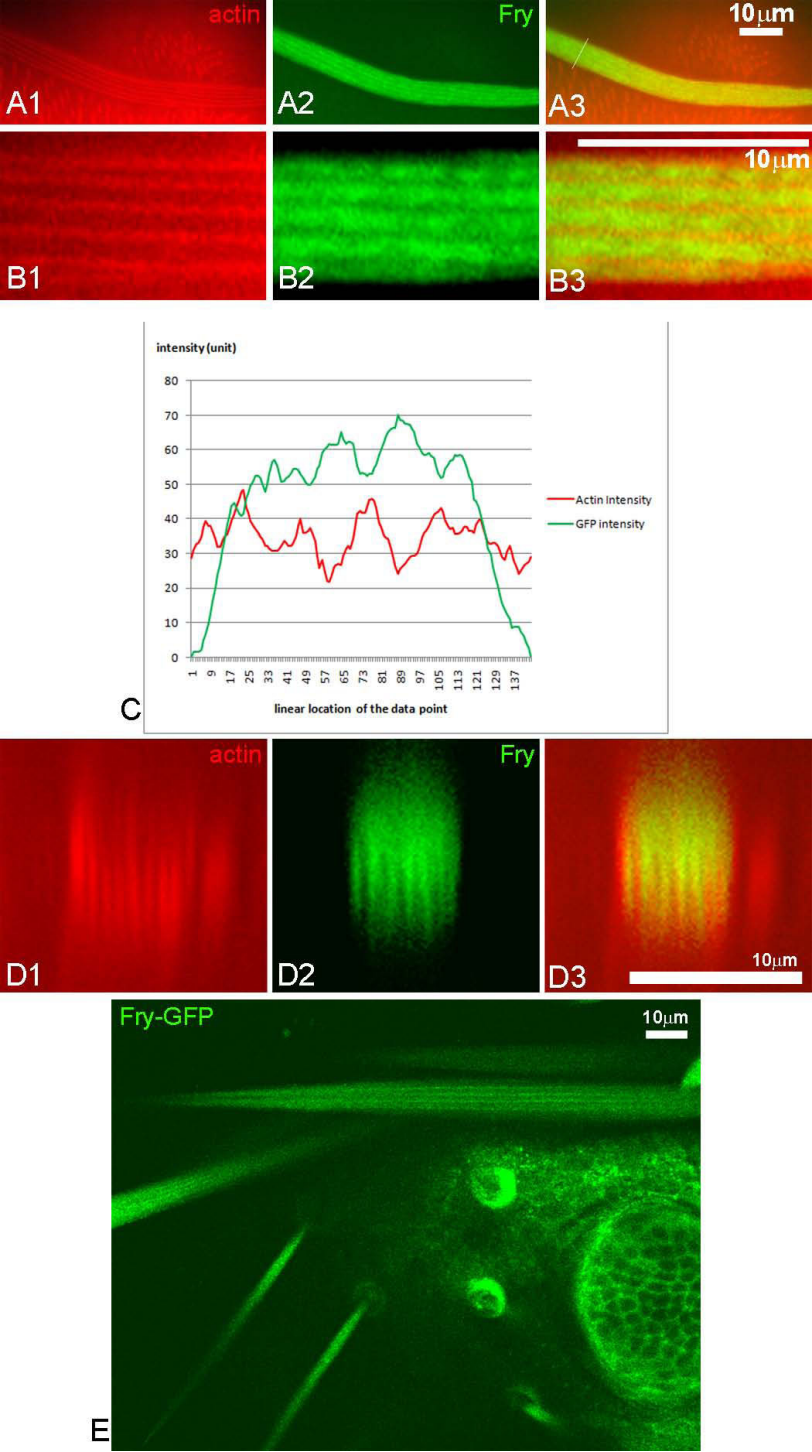
**Over-expressed Fry was found in the gaps between actin bundles**. Panels A1-3 show a *neuGAL4/UAS-myc-fry-GFP *bristle with F-actin in red (Alexa 568 with phalloidin), Fry-GFP in green (enhanced by staining with an anti-GFP antibody) and merged image. Note both F-actin and Fry-GFP are in stripes. Higher magnification images of this bristle are shown in the B panels. Note that the Fry stripes are wider than the F-actin stripes. The intensity of the red and green staining across the bristle is shown in C. The red and green peaks and troughs coincide. An oblique cross section reconstructed from a stack of images (D) shows the offset between the red and green staining by an alternative approach. The stripes of Fry-GFP could be detected by in vivo imaging of bristles in *actinGAL4/UAS-myc-fry-GFP; fry*^1^/*fry*^2 ^pupae (E).

In the experiments where Myc-Fry-GFP was over expressed the protein appeared to be present in stripes along the long axis of the bristle (Figure 4A2, B2, E). This was also seen in living pupae by direct visualization of GFP (Figure 4E), thus the stripes were not a staining artifact. Large bundles of tightly cross-linked F-actin are also found in stripes oriented along the long axis [25]. To determine the relationship between the Fry stripes and the F-actin bundles we examined bristles stained with both anti-GFP antibody and phalloidin. It was clear that the stripes of Fry staining were in between the F-actin bundles (Figure 4A-D). It is possible that the stripes were simply a consequence of Fry being excluded from the highly crossed linked bundles of F-actin.

**Figure 5:**
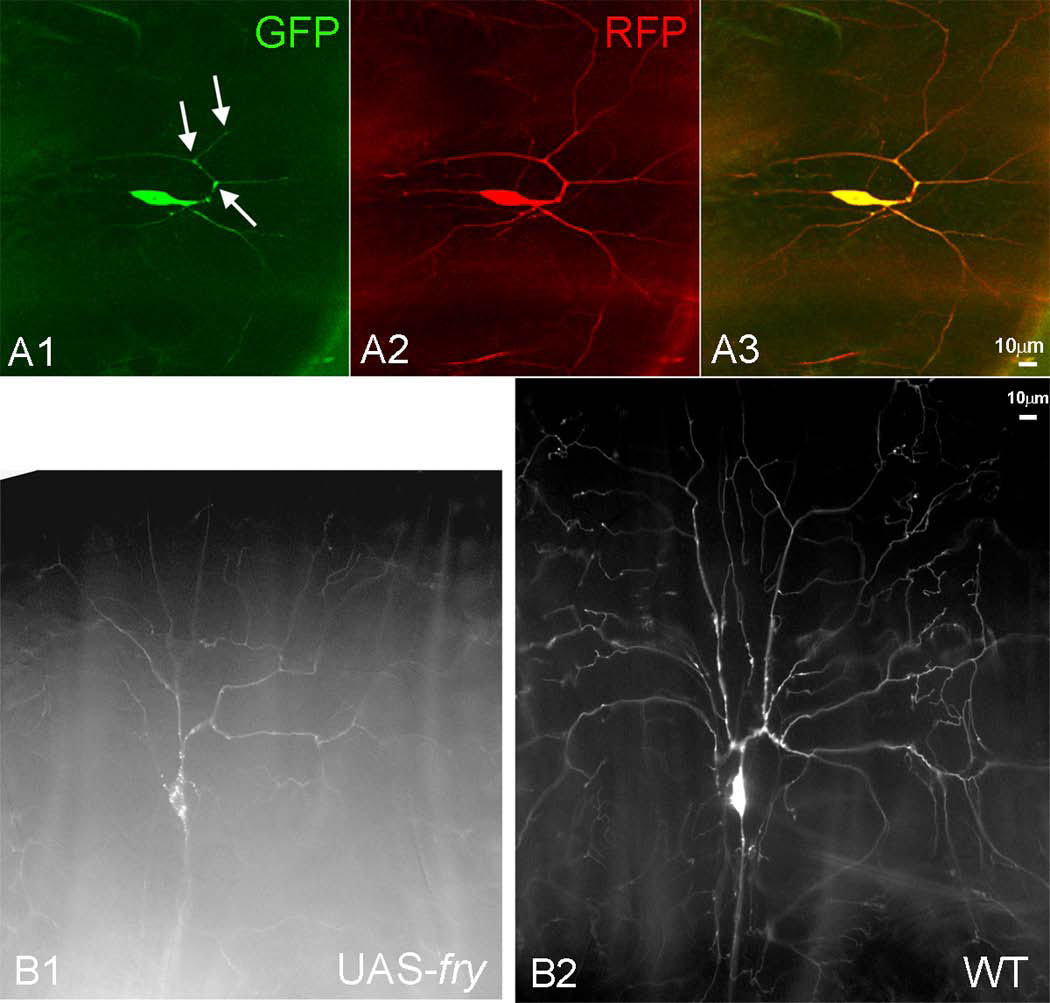
**Localization of Fry and Trc in da neurons**. Fry-GFP was imaged in living white prepupae. Single confocal optical sections are shown. A da neuron from a *ppkGAL4-UAS-mCD8RFP/UAS-myc-fry-GFP *white prepupae showed bright GFP signals at nodes (arrows in A1). The RFP signal was also strong at the nodes (A2) but the merged image showed that the distribution of the two fluorescent proteins was not equivalent. The amount of dendrite branching appeared to be decreased in *ppkGAL4-UAS-mCD8RFP/UAS-myc-fry-GFP *da neurons (B1) compared to *ppkGAL4-UAS-mCD8RFP/ppkGAL4-UAS-mCD8RFP *da neurons (B2) consistent with Fry functioning to inhibit branching.

To localize the Fry protein in da neurons we expressed *UAS*-*myc-fry-GFP *using *ppk-Gal4*. We observed the apparent preferential accumulation of Fry-GFP at nodes of dendrites of da neurons in white prepupae (wpp) (Figure 5A). However, it seemed possible that the higher GFP levels could simply be due to the nodes being thicker. To test that we co-expressed a membrane tagged red fluorescent protein (mCD8-RFP) and indeed found that it also showed higher fluorescence at the nodes (Figure 5A), however the relative intensity of RFP and GFP varied across the dendrite and some nodes were enriched for Fry compared to RFP. A more extensive quantitative analysis will be needed to determine the significance of this observation. Previously it was found that the over expression of wild type Trc led to a decrease in the da neuron dendrite arbor compared to wild type [4]. That also appeared to be the case in when Fry was over expressed (Figure 5B), but we did not quantify the phenotype.

**Figure 6:**
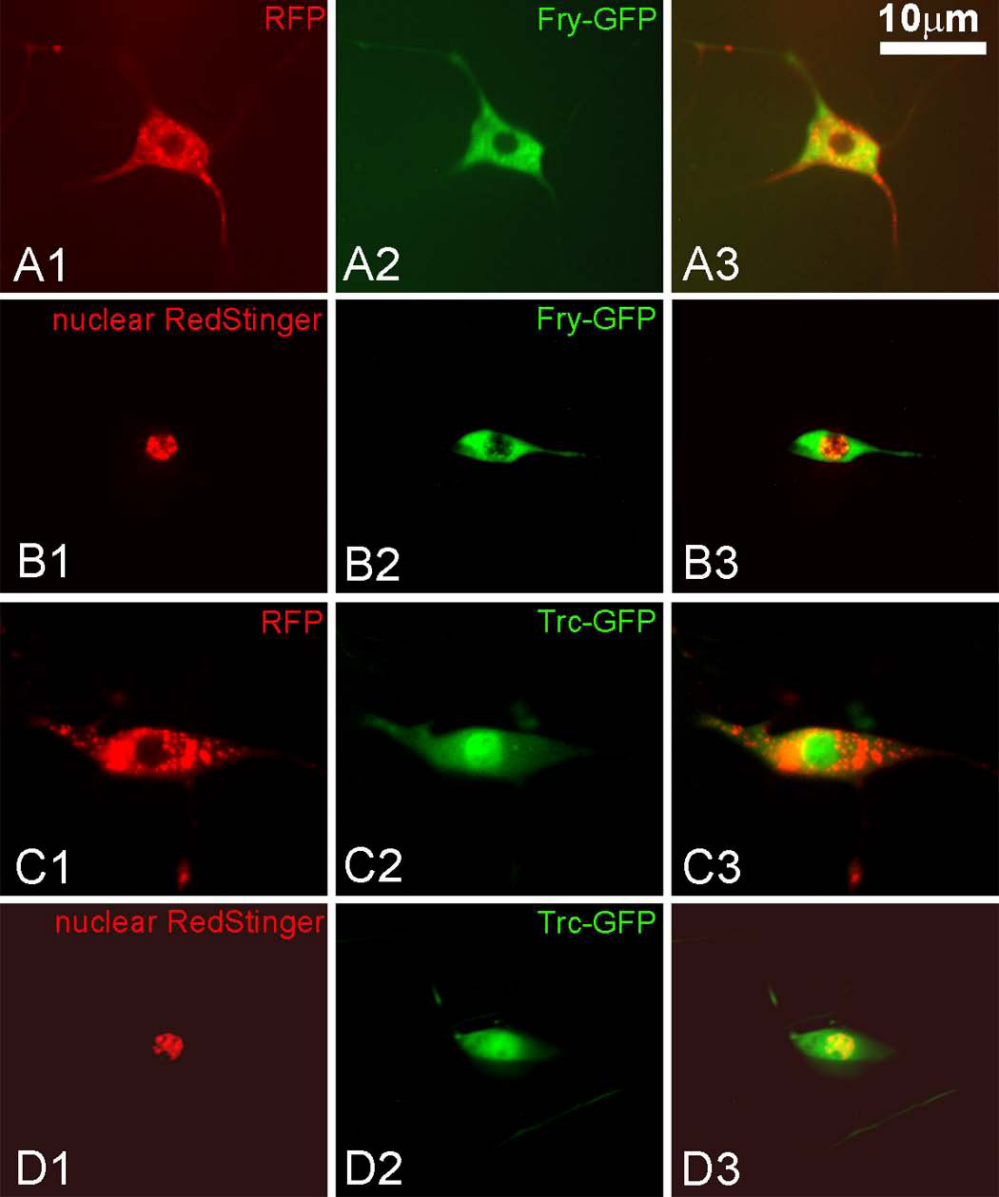
**Localization of Fry and Trc in da neurons**. Panels A1-A3 show a da neuron from a *ppkGAL4-UAS-mCD8RFP/UAS-fry-GFP *white prepupae. A1 shows cytoplasmic RFP, A2 Fry-GFP and A3 a merged image. Note that the GFP signal is excluded from nucleus, but is found throughout the cytoplasm. Panels B1-3 show a da neuron from a *ppkGAL4/UAS-fry-GFP;UAS-RedStinger/+ *white prepupae. B1 shows nuclear RFP, B2 Fry-GFP and B3 a merged image. Note that there is little overlap between the nuclear RedStinger and Fry-GFP. The modest GFP signal over the nuclear region is sometimes seen but it is not clear if this is due to overlaying cytoplasm or actual nuclear Fry. Panels C1-3 show a da neuron from a *ppkGAL4-UAS-mCD8RFP/UAS-trc-GFP *white prepupae. C1 shows cytoplasmic RFP, C2 Trc-GFP and C3 a merged image. Note that the GFP signal is brighter in the nucleus than cytoplasm. Panels D1-3 show a da neuron from a *ppkGAL4/UAS-trc-GFP;UAS-RedStinger/+ *white prepupae. In this case the RedStinger is nuclear and once again Trc-GFP is found in both the nucleus and cytoplasm.

In our initial experiments it appeared that Fry was excluded from the nucleus (Figure 6A). To confirm that the area lacking Myc-Fry-GFP was in fact the nucleus we co-expressed Myc-Fry-GFP with the nuclear marker RedStinger (Figure 6B1). In the da neurons of these animals the Myc-Fry-GFP was almost completely found in the cytoplasm even though it was being over expressed (Figure 6B2). We also examined the distribution of Trc in da neurons using a *UAS-trc-GFP *transgene. In contrast to Myc-Fry-GFP, Trc-GFP preferentially accumulated in the nuclei of da neurons (Figure 6C, D), although it was also present in the cytoplasm. In contrast, in pupal wing cells we previously found Trc to be completely cytoplasmic [10]. This demonstrated that as is the case for NDR1 in mammalian cells the cytoplasmic versus nuclear localization of Trc (NDR1 in mammals) is cell type specific. The situation in da neurons is formally the same as in yeast daughter cells where Tao3 (*fry *homolog) is cytoplasmic and Cbk1 (*trc *homolog) is nuclear [3,5].

We also generated a transgene that expressed the amino terminal 1637 amino acids of Fry tagged with an amino terminal 6XMyc (we refer to this protein as Myc-N-Fry). This fragment includes the most conserved region of Fry and was previously found to be sufficient for binding and co-immunoprecipitation with Trc [10]. The Myc-N-Fry protein showed a punctate localization in wing hairs and bristles (Figure 3D, E), but it did not extensively colocalize with endogenous Fry nor did it accumulate at the tips of bristles as endogenous Fry did (Figure 3D, E). However, Myc-N-Fry showed a high degree of colocalization with endogenous Trc. This was seen both in sensory bristles and wing hairs (Figure 3F, G). As expected Myc-N-Fry did not have any rescue activity nor did it act as a dominant negative (e.g. being expressed by a strong Gal4 driver did not result in a mutant phenotype). In addition it did not enhance or suppress the *trc*^S292AT453A ^double mutant dominant negative phenotype when both were expressed using *ptc-Gal4 *(data not shown).

Our observations indicate that the interaction of Fry and Trc, defined by both genetic and biochemical experiments, is not reflected in a consistent and complete co-localization. Rather, the limited co-localization suggests the function of the proteins only involves a transient physical interaction

### The Fry Protein is highly mobile

**Figure 7:**
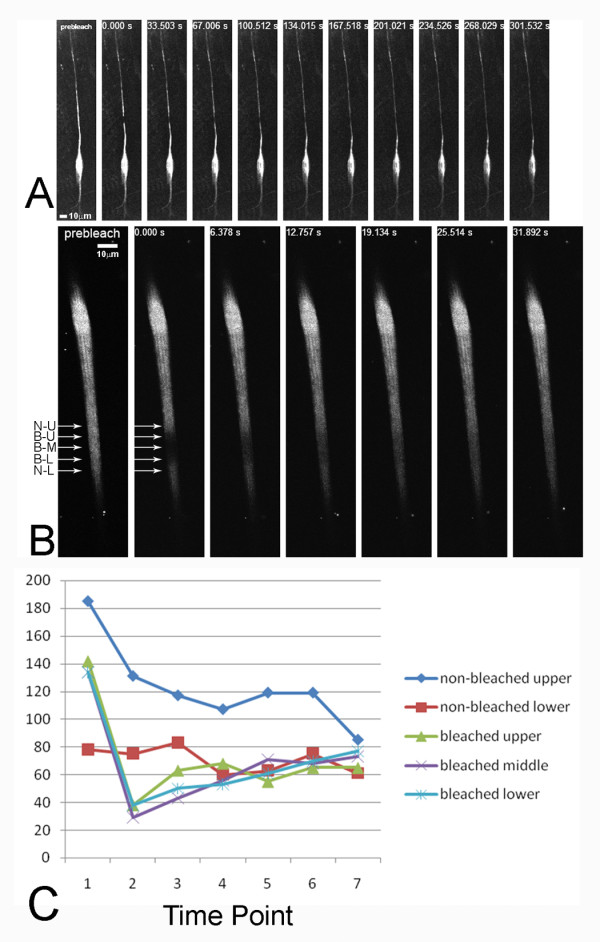
**Myc-Fry-GFP moves rapidly in da neurons and bristles**. A FRAP experiment on a single *ppkGAL4/UAS-myc-fry-GFP *da neuron is shown in A. One image is prior to bleaching. The next image is immediately after bleaching. The subsequent images were taken at 33.5 second intervals. Note that by 67 seconds substantial recovery has take place and it was essentially complete by 2 minutes. A FRAP experiment on an *actinGAL4/UAS-myc-fry-GFP; fry*^1^/*fry*^2 ^bristle. The recovery in this experiment was quite rapid and was complete by 30 seconds. N-U, non-bleached upper position; B-U, bleached upper position; B-M, bleached middle position; B-L, bleached lower position; N-L, non-bleached lower position. Panel C shows the intensity of GFP at various times at several locations in the bristle shown in Panel B. 1-7 refer to 7 different time points. 1, pre-bleach; 2, 0 second (right after bleach); 3, 6.378 seconds; 4, 12.757 seconds; 5, 19.134 seconds; 6, 25.514 seconds; 7, 31.892 seconds.

The *myc-fry-GFP *transgene allowed us to visualize Fry in living cells and we took advantage of this to assess the mobility of the protein in both bristles and dendrites of da neuron. We carried out FRAP (fluorescence recovery after photobleaching) experiments on both of these cell types. For bristles we used *UAS-myc-fry-GFP/actinGAL4; fry*^1^/*fry*^2 ^pupae. In these animals the transgene encoded Fry rescued the recessive lethality of the *fry *mutation and the only functional Fry protein was GFP-tagged. For da neuron dendrites we used *UAS-myc-fry-GFP/+; ppk-GAL4-UAS-mCD8-RFP/+ *animals. When we examined live cells, Fry-GFP appeared to be in rapidly moving small particles (Additional Files 1 (movie S1), 2 (movie S2)). When we bleached segments of either of these cell types we observed a rapid recovery of fluorescence (less than one minute) (Figure 7A, B)(Additional Files 1 (movie S1), 2 (movieS2)). The movement was rapid enough and the particles small enough that we could not follow individual particles. In experiments where we bleached the internal segment it appeared to recover from both ends (Figure 7B, C). In experiments where we bleached the distal tip of bristles it recovered from the base to the tip (Additional File 3, Figure S1). Based on these experiments, we suggest that the movement of Myc-Fry-GFP was bi-directional.

### Fry directly interacts with the C-terminal region of Trc

**Figure 8:**
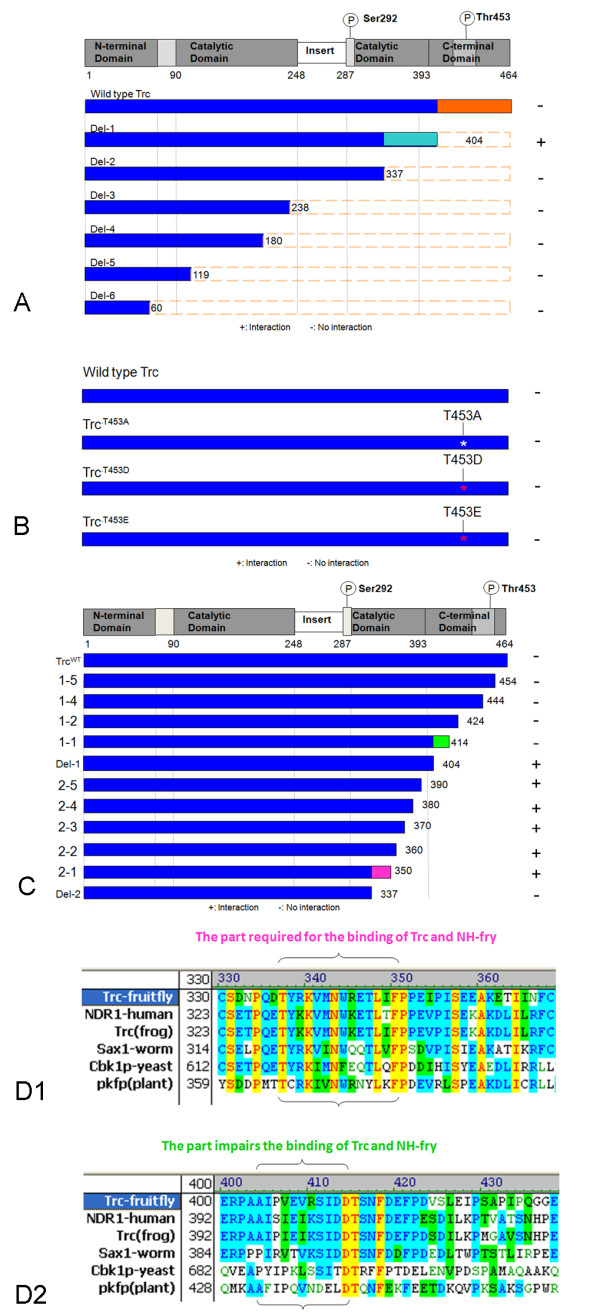
**Trc amino acids important for the interaction with Fry**. Panel A shows a diagram of the Trc protein and below in blue are the proteins (wild type and mutant) that were the initial set of deletion mutants analyzed. The interaction of these proteins with N-Fry in the yeast two hybrid system is shown. Deletion of the amino acids from 337-404 (in turquoise) resulted in a loss of the interaction while deletion of amino acids 404-464 (in orange) resulted in a stronger interaction. + indicates a positive interaction with N-Fry, and - no interaction (same meanings in B and C). Panel B shows a set of phosphorylation site mutations that were tested in the two hybrid system. The mutants did not differ from wild type Trc indicating phosphorylation of the Trc 453T did not regulate the interaction with N-Fry. In panel C a set of additional Trc deletion mutants were tested for their interaction with N-Fry in the two hybrid system. Deletion of the segment from 424 to 414 (colored in green) resulted in enhanced interaction while deletion of the segment in purple (337-350) resulted in a loss of the interaction. Panels D1 and D2 show the amino acids in these regions are are conserved in other NDR kinases.

Previous data showed that the amino terminal fragment of Fry (including the Fry domain) and Trc could be co-immunoprecipitated from *Drosophila *S2 cells [10]. We subcloned this fragment of Fry into the pGADT7 vector and used the yeast-two-hybrid system to identify the region of Trc that mediated the interaction. We first tested a series of Trc deletion mutants. Surprisingly, we found that full length Trc interacted weakly if at all with Myc-N-Fry but that a truncated Trc (amino acids 1-404) that was missing the 60 most C-terminal residues interacted strongly (Figure 7A). This interaction was lost when a smaller Trc protein (amino acids 1-337) was used (Figure 8A). Thus, residues from the carboxy terminal region of Trc inhibited the two-hybrid interaction. One possibility was that in vivo the interaction of Fry and Trc is regulated by the phosphorylation of the C-terminal hydrophobic motif site (T453). To test this hypothesis we used mutant Trc proteins in the 2-hybrid system. We tested the interaction between Myc-N-Fry and trc^T453A ^(dominant negative construct), trc^T453D ^(constitutively active construct) or trc^T453E ^(a second constitutively active construct) and found no difference compared to wild type Trc (Figure 8B). We therefore concluded that phosphorylation at T453 did not regulate the interaction between Trc and Fry. Another possibility to explain the lack of a strong 2 hybrid interaction between Trc and Myc-N-Fry is that a third Drosophila protein is part of the complex in the fly and in its absence the Trc carboxy terminal region inhibits the interaction. A Mob protein would be a candidate to be involved in such an interaction as Mob and Trc are known to interact. To further map the location of residues that inhibited the interaction with Myc-N-Fry we made a further set of Trc deletions and used them in the two-hybrid system (Figure 8C). We found that extending the deletion from amino acid 414 to 404 led to a strong interaction with Myc-N-Fry (Figure 8C) implicating residues from this region as being important. This 11 aa region is highly conserved with 7/11 residues identical between the fly, frog and human homologs and two of the differences are conservative (V-I, R-K) substitutions (Figure 8D2). Control western blot experiments established that the non-interacting mutant proteins were expressed (Additional File 4, Figure S2).

The data from our original set of Trc deletions indicated that residues from 338-404 were essential for the interaction. We generated and tested additional deletions and found that extending the deletion from aa 350 to 337 abolished the interaction (Figure 7C). These residues could contain the binding surface for the interaction or be required for the folding of the protein to allow other residues to bind to Myc-N-Fry. This region is within the catalytic domain (sub-domain X) [26], and based on the 3-D structure of the related AGC family kinase human Sgk, this region is exposed and available for interacting with other proteins (the 3D structure of Sgk is available at http://www.ncbi.nlm.nih.gov/Structure/mmdb/mmdbsrv.cgi?uid=75006). The region is highly conserved with 11/14 residues being identical between the fly, frog and human Trc/NDR proteins and one of the differences is conservative (R-K) Figure 7D1).

**Figure 9:**
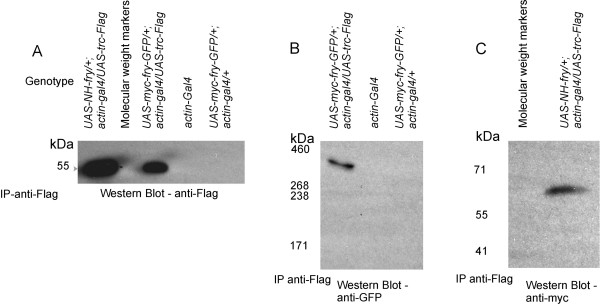
**Fry and N-Fry co-immunoprecipitate with Trc-FLAG**. A. Shows the detection by mouse anti-Flag of the proteins pulled down by rabbit-anti-FLAG antibody. The Trc-FLAG fusion protein is approximately 52 kDa, and is only detected when both *UAS-trc-FLAG *and *actin-GAL4 *are present. B. Full-length Fry co-immunoprecipitated with Trc-FLAG. Trc-FLAG was pulled down using rabbit-anti-FLAG antibody and the Western Blot was probed using mouse-anti-GFP antibody. Full-length Fry-GFP (418 kDa) is detected only from extracts made from discs that expressed *UAS-trc-FLAG *and *UAS-myc-fry-GFP *together by *actin-GAL4*. C. Myc-N-Fry co-immunoprecipitated with Trc-FLAG (pulled down by rabbit-anti-FLAG antibody and detected by mouse-anti-Myc antibody) (size of the protein detected by the anti-myc antibody was smaller than the predicted size of Myc-N-Fry, which should be ~180 kDa. A band of the same size was also detected in western blots of extracts of discs that expressed Myc-N-Fry and it is likely to be a degradation product.

Previous experiments in the Drosophila system showed that Trc and Myc-N-Fry could be co-immunoprecipitated from S2 cells. We extended these experiments to see if either or both Myc-N-Fry or Myc-Fry-GFP would co-immunoprecipitate from wing discs or whole larvae. We used transgenic flies expressing both *UAS-myc*-*fry-GFP *and *UAS*-*trc-FLAG*, or both *UAS- Myc-N-fry *and *UAS*-*trc-FLAG *using the *actin-GAL4 *driver. We found that both Myc-N-Fry and Myc-Fry-GFP co-immunoprecipitated with Trc (Figure 9).

## Discussion and Conclusions

### The interaction of Fry and Trc

Data from a number of systems from fungi to mammalian cells indicate that the Fry and NDR kinase family proteins interact directly in vivo. The data reported here provide further support. We first established that Fry existed as a full-length protein in vivo in wing discs. We also demonstrated that the full length Fry protein could be co-immunoprecipitated with Trc from wing disc cells and using the two-hybrid system we mapped a region in the Trc protein that was essential for the interaction. We also found that a Trc protein that was missing its most C terminal region interacted more strongly with Fry than did the complete Trc protein. This suggested that the interaction was regulated and this could be an important regulatory step in vivo.

Although the Trc and Fry proteins appeared to interact in vivo we did not see extensive co-localization of the two proteins by immunostaining (Figure 3H). This was true for both the endogenous proteins and for transgene encoded proteins. In the cells we examined both proteins were distributed in a punctate manner but relatively few of the puncta stained positively for both Trc and Fry. We also found that in two cell types the two proteins had distinctly different protein accumulation patterns. In bristle forming cells the provocative distal tip accumulation of Fry was not seen with Trc. Further, in da sensory neurons Trc accumulated preferentially in nuclei, while Fry was excluded from the nucleus. We cannot rule out the possibility that there could be multiple protein complexes that contain Fry and Trc and that in some of these one or the other of the proteins is shielded from our antibody reagents. However, our data did not support this possibility. We saw extensive co-localization of the amino and carboxyl end tags of transgene encoded Fry. Thus, both ends of the Fry protein appear to be equally accessible. We also saw a high degree of co-localization between Trc and Myc-N-Fry. Thus, in any puncta that contains these proteins either both or neither are accessible to antibody. We suggest that the interaction between Fry and Trc is transient and regulated and that much of these proteins in cells are not in a complex together. Our observation that Trc accumulated in da neuron nuclei while Fry was excluded is reminiscent of the observation that CBK1p accumulated in yeast daughter cell nuclei, while Tao3p is excluded from the nucleus [3,5]. Similarly, our observations that Trc is primarily cytoplasmic in some cell types (e.g. pupal wing cells) and primarily nuclear in others (da neurons) mirror the observations that mammalian NDR1 is cytoplasmic in some cells and nuclear in others. The subcellular localization of Trc and Fry could be different in different cell types due to differential modification or to it being bound by different partners in different cell types. Trc is known to be phosphorylated and Fry protein was found to be multiply phosphorylated in embryos [10,27,28] and that seems likely in other cell types as well. Differential phosphorylation could regulate the subcellular localizations of these two proteins, although there is no direct evidence for this.

### Fry is mobile

The generation of a transgene that encoded a functional Fry-GFP protein allowed us to assess the mobility of Fry in living cells. We used FRAP to test this in both developing bristles and in the dendrites of da neurons. In both of these cell types we found Fry-GFP moved rapidly. Our data suggested that Fry-GFP moved both distally and proximally in developing bristles and dendrites of da neurons. These observations suggested that Fry was in some way involved in intracellular transport and in that way regulated polarized growth. In mammalian cells Fry has been found to bind to microtubules and such a molecular activity would nicely fit with it regulating intracellular transport.

## Methods

### Construction of full length *fry *minigene and Myc-N-*fry *cDNA

A 4.9 kb (#1-4911 bp) fragment that encoded the amino terminal part of the protein with an amino terminal Myc tag (Myc-N-Fry) was synthesized de novo. This fragment also contained several unique restriction sites to facilitate further manipulation. We obtained two middle fragments of *fry *cDNA sequences (#4810-6540 bp and #6472-8319 bp) by RT-PCR using adult mRNA as a template. The most C-terminal fragment consisted of a region from the *fry *genomic DNA (including introns) was generated by PCR. The four fragments were inserted one by one into pENTR vector (connected by BlpI, KpnI and SalI restrictional sites), and then the whole minigene was inserted into pTWG vector by enzyme-induced direct recombination in GATEWAY system (Invitrogen Inc.). Details will be provided upon request.

### Primers used for making truncated Trc constructs

We used the following PCR primers. 5' primer: 5'-GGGAATTCCATATGCACCATCACCATC-3' (with a unique NdeI site); 3' primers (with a unique EcoRI site): trc^D1-1 ^[trc cDNA #1-#1242nt, amino acid #1-414]: 5'-CCGGAATTCTCAC TCACGTATGTGCTC-3'; trc^D1-2 ^[trc cDNA #1-#1272nt, amino acid #1-424]: 5'-CCGGAATTCTCAGCGCACCTCAACGGG-3'; trc^D1-4 ^[trc cDNA #1-#1332nt, amino acid #1-444]: 5'-CCGGAATTCTCACGATGGTATCTCCAG-3'; trc^D1-5 ^[trc cDNA #1-#1362nt, amino acid #1-454]: 5'-CCGGAATTCTCACGCAATCTCACCG CC-3'; trcD^2-1 ^[trc cDNA #1-#1050nt, amino acid #1-350]: 5'-CCGGAATTCTCAG TCCTGGGGATTGTC-3'; trc^D2-2 ^[trc cDNA #1-#1080nt, amino acid #1-360]: 5'-CCGGAATTCTCACTCGCGCCAGTTCAT-3'; trc^D2-3 ^[trc cDNA #1-#1110nt, amino acid #1-370]: 5'-CCGGAATTCTCATATGGGGATCTCTGG-3'; trc^D2-4 ^[trc cDNA #1-#1140nt, amino acid #1-380]: 5'-CCGGAATTCTCAGTTGATGATCGTC TC-3', trc^D2-5 ^[trc cDNA #1-#1170nt, amino acid #1-390]: 5'-CCGGAATTCTCAA CCCAGCCGGCGATC-3'. To insert those into the pGBKT7 vector, we modified the original vector and inserted a DNA sequence containing - NdeI-KpnI-NcoI-EcoRI- XmaI-BamHI-SalI-SacI-PstI-NotI- into the multiple cloning sites.

### Oligo primers used for *trc *insertion into GATEWAY system

5'-GGGGACAAGTTTGTACAAAAAAGCAGGCTGTATGATGAGCAGCAGAACGCAGG-3' (forward) and 5'-GGGGACCACTTTGTACAAGAAAGCTGGGT TTCACTCCAAATTTCGCACCTCGA-3' (reverse) and inserted into pDEST vectors by BP reaction (Gateway technology, Invitrogen).

### Fly stocks

The *UAS-myc-fry-GFP *and *UAS-Myc-N-fry *transgenic lines were generated by embryo injection with corresponding constructs and were isolated afterwards (the injection was done by Genetics Services, Inc.). The *UAS-RedStinger *stocks were obtained from Drosophila Stock Center in Bloomington (stock number #8546 and 8547). The *UAS-trc-FLAG *[4], *UAS*-*trc*^S292AT453A ^[10]double mutant stocks, *fry*^1^/*TM6 *and *fry*^2^/*TM6 *stocks [23] were described in previous publications. Both *fry*^1 ^(frameshift mutation) and *fry*^2 ^(nonsense mutation) are null alleles. *ppkGAL4*[27] and *ppkGAL4 UAS-mCD8RFP *stocks were constructed in the Jan Lab at UCSF and have been extensively characterized [4]. The *ppkGal4 *gene drives expression in the da neurons.

### Microscopy

A Spot digital camera (National Diagnostics, Manville, NJ) on a Zeiss Axioskop microscope (Thornwood, NY) was used to obtain the bright field images. For most experiments the samples were examined using an Atto spinning disc confocal attachment on a Nikon microscope. The MetaMorph software (Molecular Device, MDS Inc.) was used to take the individual images and Z-stacks. Some images were obtained using a Biorad Radiance 2100 laser scanning confocal microscope or on a ZEISS510 spectral confocal microscope at the Keck Center for Cellular Imaging at the University of Virginia. The Image J software (Wayne Rasband, National Institutes of Health, USA) was used to analyze actin or GFP intensity. Statistical analysis of the data was done in Microsoft Excel (Microsoft).

### Immunohistochemistry

The larvae or pupae were dissected in PBS, fixed in 4% paraformaldehyde/PBS for 3 hours, and the wing discs/pupal wings were dissected in PBS. After 1-hour incubation in 10% goat serum/PBST, the tissues were incubated with the primary antibodies at 4°C overnight. Then the tissues were rinsed in PBST for three times (15 minutes each), incubated in 10% goat serum/PBST for an hour, and then incubated with the secondary antibodies. Finally the tissues were rinsed in PBST for three times (10 minutes each), then rinsed in PBS, and mounted in the Prolong antifade mounting medium (Invitrogen). All the steps were at room temperature unless specifically mentioned.

### Immunoprecipitation and Western blot

Immunoprecipitation Kits (protein A and protein G) (Roche) were used. For pull-down experiment, the antibody used was 6 μl/ml protein solution; for detection, the primary antibody dilution in PBSTM was 1:3000 (except for mouse-anti-Myc antibody, which was 1:500) and the secondary antibody dilution in PBSTM was 1:5000. NuPAGE large protein Analysis System (Invitrogen Corp.) and common western kit (Novex 4-20% Tris-Glycine gel, running buffer, transfer buffer, PVDF membrane, etc. were obtained from Invitrogen) were used.

### Yeast two-hybrid system

The Matchmaker GAL4 two hybrid system 3 (Clontech) was used. For a pair of proteins to be scored as interacting we required complementation for growth and for the induction of expression of β-Galactosidase.

### FRAP (fluorescence recovery after photobleaching)

FRAP studies were performed on live animal using a ZEISS510 confocal/Spectral microscope with a 63× objective (for experiments on bristles) or with a 25× objective (for experiments on da neurons). Sampling rate was 3 s per time point. The actual bleach time varies according to the bleached area for each sample at high laser intensity. To study the Fry movement in da neurons, we crossed flies *ppkGAL4 UASmCD8RFP/TM6 *with *UAS-myc-fry-GFP/UAS-myc-fry-GFP *and observed *ppkGAL4 UASmCD8 RFP/UAS-myc-fry-GFP *flies. To study Fry movement in bristles, we crossed *actin-GAL4/CyO*; *fry*^1^/*TM6 *flies with *UAS-myc-fry-GFP/UAS-myc-fry-GFP; fry*^2^/*TM6 *flies and observed *actin-GAL4/UAS-myc-fry*;*fry*^1^/*fry*^2 ^flies. Before live imaging, white prepupae (for da neuron experiments) were washed in 50% bleach solution. The 44-48-hour APF pupae used for bristle observation were washed, placed in an observation chamber and their thorax revealed by creating a window in the pupal case by dissection.

### Antibodies

Anti-Trc polyclonal antibody was generated by immunizing rat with the full-length Trc-GST fusion protein purified from Escherichia coli, which was transformed with wild type trc cDNA. The anti-Fry antibody was also developed in our laboratory and has been described previously [10]. Mouse anti-Myc (Cat#R950-25), mouse anti-Myc-HRP antibody (Cat#R951-25), rabbit anti-GFP (Cat#A-11122), phalloidin 568 (Cat#A12380), anti-rabbit-488 (Cat#A-11008), anti-mouse-488 (Cat#A-11001), anti-mouse-568 (Cat#A11004), and anti-rat-568 (Cat#A-11077) were obtained from Invitrogen Corp. Rabbit-anti-FLAG (Cat# F7425) and mouse-anti-FLAG (Cat# F1804) were obtained from Sigma. Mouse-anti-GFP was obtained from Clontech (Cat# 632381). HRP-anti-mouse (Cat# T-20914) was obtained from Molecular Probes.

## Abbreviations

*fry: furry*; NDR: nuclear DBF2 related; GFP: Green Fluorescent Protein; *trc*: *tricornered*; FRAP: fluorescence recovery after photobleaching; wpp: white prepupae; SGK: serum, glucocorticoid regulated kinase; RFP: red fluorescent protein; PBS: phosphate buffered saline; SEM: standard error of the mean.

## Authors' contributions

XF carried out most of the experiments including the assembly of the Fry transgene, the two hybrid analysis of the Trc - Fry interaction, the genetic rescue experiments on the *fry *transgene, the biochemical experiments on the Fry protein, the immunostaining and in vivo imaging experiements. XF also played a large role in writing the paper. KE generated cDNAs for the middle section of the *fry *mRNA that were used for constructing the transgene. QL carried out several of the yeast and western blot experiments. PNA supervised the overall project, carried out a few of the genetic and immunostaining experiments and also played a role in writing the paper. All of the authors read and approved the manuscript.

## Supplementary Material

Additional file 1 **Movie S1. FRAP of Fry-GFP in a bristle**. The bristle was bleached in an intermediate position and followed during recovery. Click here for file

Additional file 2 **Movie S2. FRAP of Fry-GFP in a da neuron dendrite**. The dendrite was bleached distally and followed during recovery. Click here for file

Additional file 3 **Figure S1.  Distal bleaching of Myc-Fry-GFP in growing bristles**. A. A *UAS-myc-fry-GFP; neur-Gal4/+ *pupal bristle was bleached at its distal tip and then followed by time lapse confocal microscopy. B. The GFP intensity was determined at the 4 locations indicated in A and plotted as a function of time. Images were obtained every 7 seconds. Click here for file

Additional file 4 **Figure S2.  Detection of the expression of DBD-trc fusion proteins in yeast cells**. The upper diagram shows a set of Trc deletion mutants that were tested for their interaction with NH-Fry in the two-hybrid system. + indicates a positive interaction with NH-Fry, and - no interaction. The lower panels show the Western blotting detection of the expressed fusion proteins of DNA-binding domain (DBD, which contains ~147 amino acids) and truncated trc proteins, linked by a short linker (which contains ~27 amino acids, including a c-Myc epitope). Mouse anti-c-Myc antibody was used to detect truncated Trc proteins. Bands of expected molecular weight are detected in all the transformed yeast cells. Tubulin level (detected by mouse anti-tubulin) was used as a loading control. Click here for file
