# Supplementary figures and images for: The Drosophila Fry protein interacts with Trc and is highly mobile in vivo
Source: BMC Dev Biol. 2010 Apr 20;10:40. doi: 10.1186/1471-213X-10-40 (PMC2868802; doi:10.1186/1471-213X-10-40)

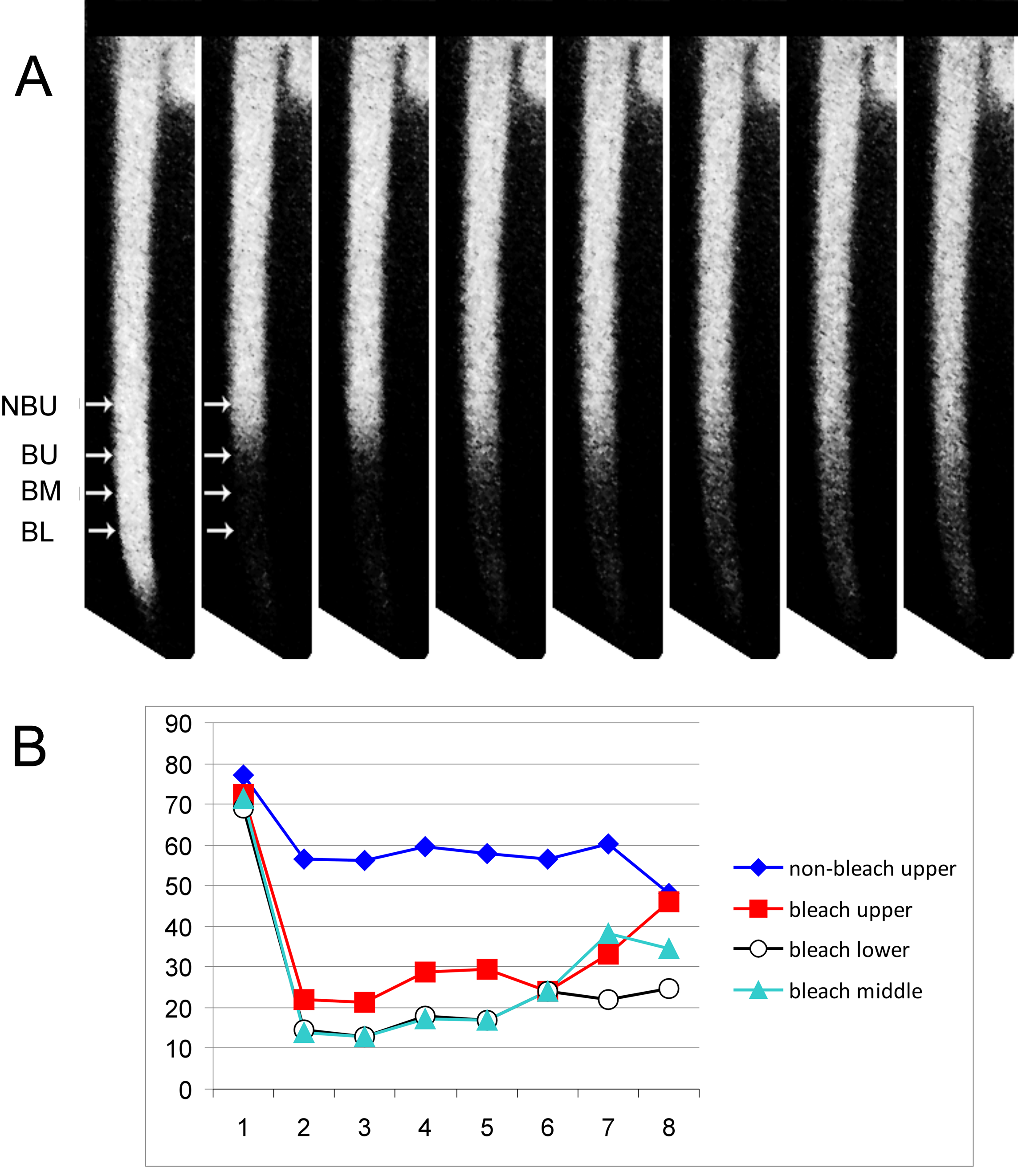

Supplement: Additional file 3 — Figure S1. Distal bleaching of Myc-Fry-GFP in growing bristles. A. A UAS-myc-fry-GFP; neur-Gal4/+ pupal bristle was bleached at its distal tip and then followed by time lapse confocal microscopy. B. The GFP intensity was determined at the 4 locations indicated in A and plotted as a function of time. Images were obtained every 7 seconds. [file 1471-213X-10-40-S3.TIFF]

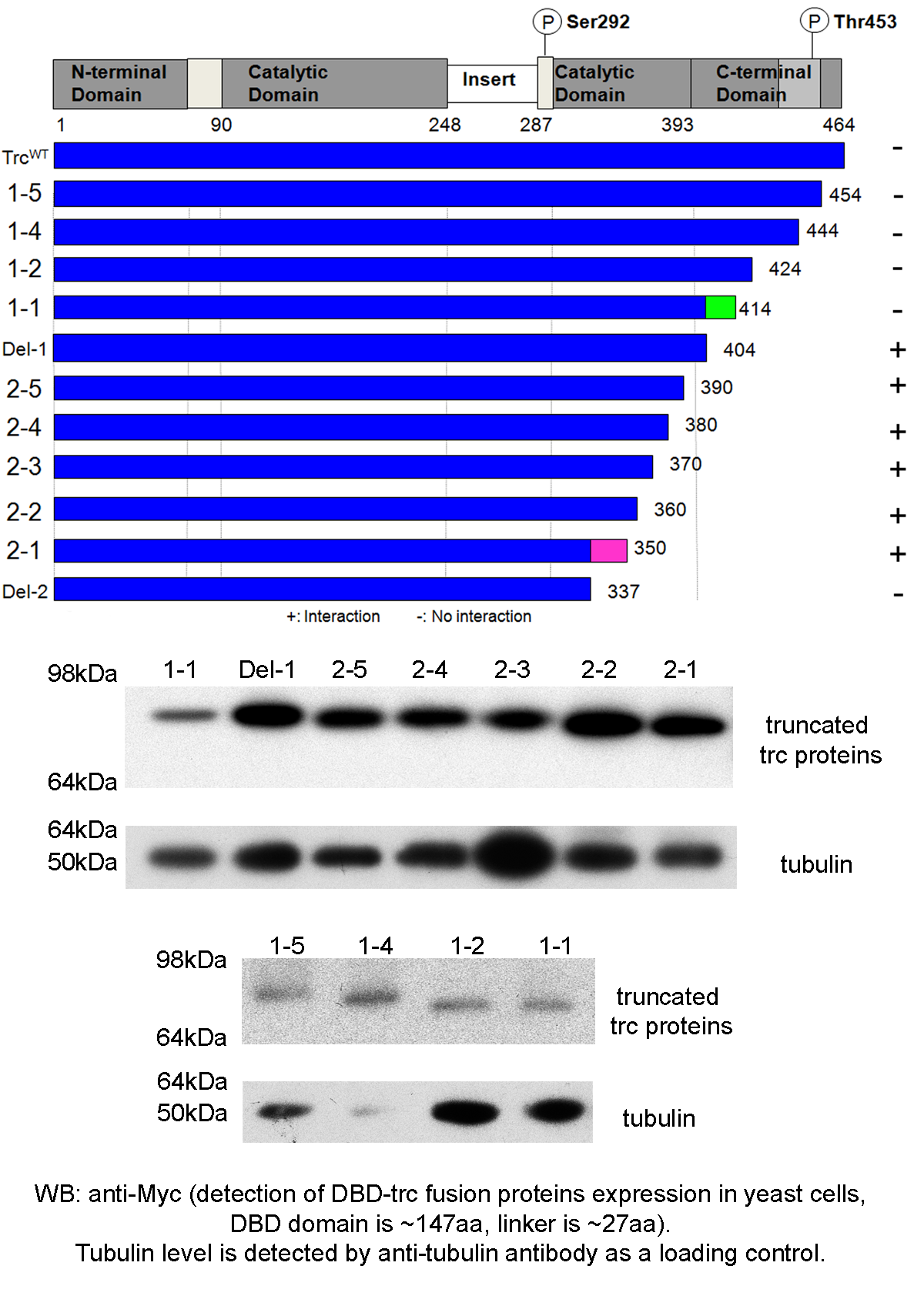

Supplement: Additional file 4 — Figure S2. Detection of the expression of DBD-trc fusion proteins in yeast cells. The upper diagram shows a set of Trc deletion mutants that were tested for their interaction with NH-Fry in the two-hybrid system. + indicates a positive interaction with NH-Fry, and - no interaction. The lower panels show the Western blotting detection of the expressed fusion proteins of DNA-binding domain (DBD, which contains ~147 amino acids) and truncated trc proteins, linked by a short linker (which contains ~27 amino acids, including a c-Myc epitope). Mouse anti-c-Myc antibody was used to detect truncated Trc proteins. Bands of expected molecular weight are detected in all the transformed yeast cells. Tubulin level (detected by mouse anti-tubulin) was used as a loading control. [file 1471-213X-10-40-S4.TIFF]
